# E-cadherin deficiency promotes prostate macrophage inflammation and bladder overactivity in aged male mice

**DOI:** 10.18632/aging.203994

**Published:** 2022-03-31

**Authors:** Laura E. Pascal, Taro Igarashi, Shinsuke Mizoguchi, Wei Chen, Lora H. Rigatti, Caroline G. Madigan, Rajiv Dhir, Wade Bushman, Donald B. DeFranco, Naoki Yoshimura, Zhou Wang

**Affiliations:** 1 Department of Urology, University of Pittsburgh School of Medicine, Pittsburgh, PA 15232, USA; 2 Department of Pharmacology and Chemical Biology, University of Pittsburgh School of Medicine, Pittsburgh, PA 15232, USA; 3 UPMC Hillman Cancer Center, University of Pittsburgh School of Medicine, Pittsburgh, PA 15232, USA; 4 Division of Laboratory Animal Resources, University of Pittsburgh School of Medicine, Pittsburgh, PA 15232, USA; 5 Department of Pathology, University of Pittsburgh School of Medicine, Pittsburgh, PA 15232, USA; 6 Department of Urology, University of Wisconsin, Madison, WI 53705, USA; 7 Pittsburgh Institute for Neurodegenerative Diseases, University of Pittsburgh School of Medicine, Pittsburgh, PA 15232, USA

**Keywords:** Cdh1, aging, E-cadherin, prostatic inflammation, BPH, bladder overactivity, LUTS

## Abstract

Decreased E-cadherin immunostaining is frequently observed in benign prostatic hyperplasia (BPH) and was recently correlated with increased inflammation in aging prostate. Homozygous E-cadherin deletion in the murine prostate results in prostate inflammation and bladder overactivity at 6 months of age. However, this model is limited in that while E-cadherin is significantly reduced in BPH, it is not completely lost; BPH is also strongly associated with advanced age and is infrequent in young men. Here, we examined the functional consequences of aging in male mice with prostate luminal epithelial cell-specific E-cadherin heterozygosity. In control mice, aging alone resulted in an increase in prostate inflammation and changes in bladder voiding function indicative of bladder underactivity. At 24 months of age, mice with prostate-specific Cre-mediated heterozygous deletion of E-cadherin induced at 7 weeks of age developed additional prostatic defects, particularly increased macrophage inflammation and stromal proliferation, and bladder overactivity compared to age-matched control mice, which are similar to BPH/LUTS in that the phenotype is slow-progressing and age-dependent. These findings suggest that decreased E-cadherin may promote macrophage inflammation and fibrosis in the prostate and subsequent bladder overactivity in aging men, promoting the development and progression of BPH/LUTS.

## INTRODUCTION

Benign prostatic hyperplasia (BPH) is a common age-related disease in men and is frequently associated with burdensome lower urinary tract symptoms (LUTS) [1]. Histologically, BPH is characterized by benign, nodular proliferation of prostate glands and/or stromal cells in the central or transition zone of the prostate; however, histologic BPH may or may not be symptomatic and men can develop LUTS in the absence of histologic BPH [1]. Although the etiology is not well understood, frequently associated risk factors for BPH/LUTS include aging [2–4] and prostatic inflammation [5]. We recently reported that the aging prostate is subject to increased prostatic inflammation and decreased E-cadherin and that E-cadherin is further decreased in BPH tissues [6], suggesting that aging could contribute to the increased prostatic inflammation and reduction in E-cadherin observed in BPH tissues.

Aging-related changes that are associated with BPH/LUTS have also been reported in mice, including an increase in prostatic inflammation and altered urinary function. Mice at 12 months of age have an increase in inflammatory infiltrates and increased vascularization compared to mice at 2 months of age [7, 8]. At 24 months of age, the stromal compartment of murine prostate exhibits increased collagen deposition, disorganization of smooth muscle cells and extra-cellular matrix and an increase in inflammatory infiltrates – particularly T and B cells - compared to young animals [9]. Genes associated with extracellular matrix, cell adhesion and focal adhesion are down-regulated in aged stromal and epithelial compartments in both the human and murine prostates [8, 10]. Previous comparison studies of aged versus young mice revealed changes in bladder voiding function changes, including reduced bladder contractility and smaller voided volumes per micturition in both male and female aged mice [11, 12]. Collectively, these studies suggest that similar to humans, the aging mouse undergoes lower urinary tract changes including prostatic stromal fibrosis and inflammation as well as changes in bladder function.

We recently generated a murine model with homozygous deletion of the E-cadherin gene in prostatic luminal epithelial cells to determine functional consequences of E-cadherin ablation in BPH pathogenesis [13]. While these mice developed prostatic inflammation and overactive bladder at 6 months of age [13], this does not match strong aging-dependent development of LUTS in humans. This may be related to the findings that E-cadherin is only partially reduced in BPH luminal cells [14–16] and aging alone may induce changes in prostate and bladder [11, 12]. Thus, to model the partial loss of E-cadherin expression on LUTS during aging in humans, we examined lower urinary tract function and pathology in aged transgenic mice with heterozygous deletion of E-cadherin in prostate luminal epithelial cells. Aged control mice displayed an increase in prostatic inflammation and changes in bladder voiding characteristics compared to control mice at 6 months of age, while aged mice with heterozygous deletion of E-cadherin displayed an increase in prostate macrophages, stromal proliferation and fibrosis, and bladder overactivity compared to age matched controls. Taken together, these findings suggest age-dependent changes induced by E-cadherin heterozygosity in the murine prostate could promote a BPH/LUTS-like phenotype in the prostate and bladder.

## RESULTS

### E-cadherin in human BPH and transgenic mouse prostate

**Figure 1:**
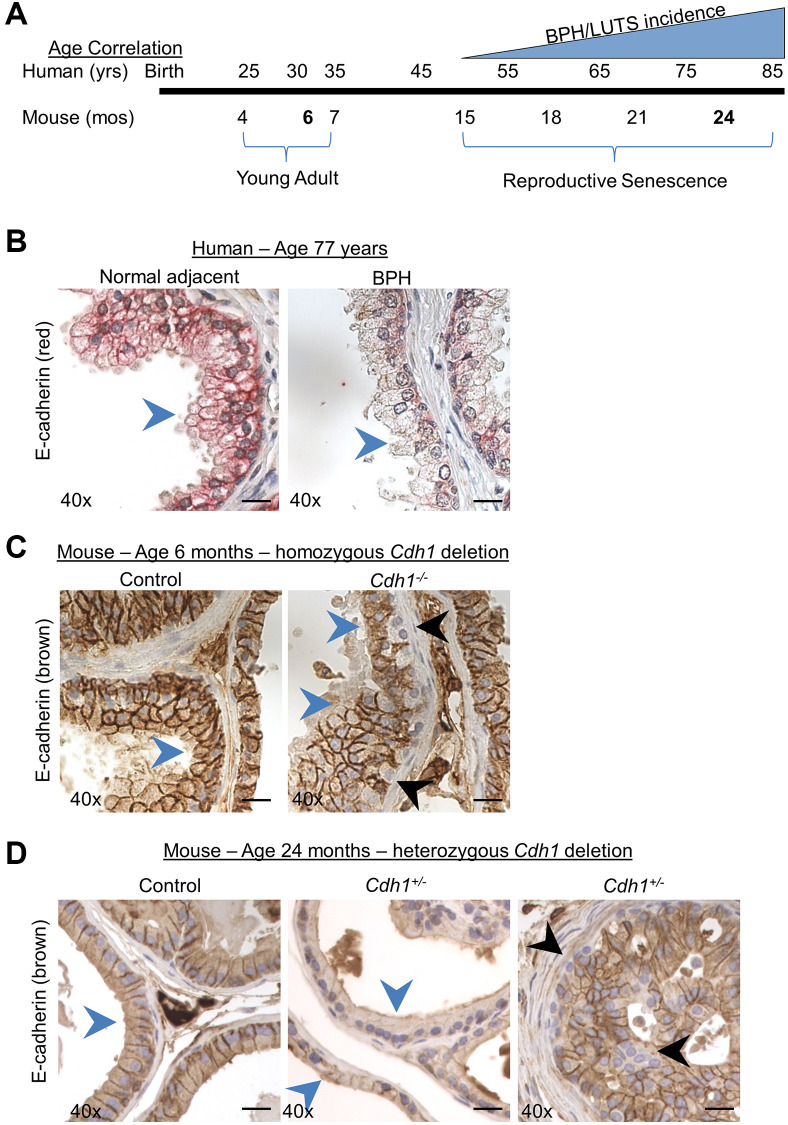
**Modeling the combined impact of heterozygous E-cadherin loss and aging on the murine prostate.** (**A**) Correlation of human age and BPH/LUTS incidence to aging of C57BL/6 mice [18]. Experimental timepoints for mice at 6 months and 24 months of age (noted in bold) correlate to 30 years and 75–85 years of age for men. (**B**) Representative E-cadherin immunostaining (red) in normal prostate adjacent to BPH (left) and in glandular BPH (right) specimens from a 77-year-old patient. (**C**) E-cadherin immunostaining (brown) in Control (left) and mice with homozygous deletion of E-cadherin (*Cdh1*^-/-^) (right) mouse ventral prostate at 6 months of age. E-cadherin immunostaining in *Cdh1*^-/-^ mice displayed a mosaic pattern of E-cadherin negative cells (black arrows) surrounded by a layer of E-cadherin positive epithelial cells (blue arrows). (**D**) E-cadherin immunostaining in Control (left) and mice with heterozygous deletion of E-cadherin (*Cdh1*^+/-^) mouse ventral prostate (center showing apparent reduced E-cadherin immunostaining, and right panel showing mosaic staining pattern) at 24 months of age. Blue arrows indicate epithelial cells with positive E-cadherin staining, black arrow indicates E-cadherin negative epithelial cells. Original magnification, 40×. Scale bars indicate 25 μm.

BPH and LUTS are typically associated with aging, and seldom identified in patients under the age of 51 [2, 3, 17]. In a recent study of the impact of age on the lower urogenital tract in male mice, aging mice displayed prostatic enlargement and altered urinary function compared to young animals [11], illustrating the potential relevance of an aging rodent model to identify mechanisms driving BPH/LUTS in humans. Mice reach adulthood at approximately 10 weeks of age and undergo reproductive senescence from 15–18 months of age, while post 18 months of age is considered to be the post-reproductive senescence period for mice [18]. The correlate to human adulthood is 20–51 years of age, reproductive senescence from 51–78 years of age and post-reproductive senescence period over age 78 [18]. Since BPH/LUTS incidence increases from ~ 50% in men at the age of 50 and reaches approximately 80% in men over the age of 70 [17], we chose to examine the impact of E-cadherin loss in male mice at 24 months of age – corresponding to 75–85 years of age in humans (Figure 1A). Previous studies examining the role of aging in the murine prostate have also examined mice at 24 months of age [9, 11]. To examine the impact of aging, we compared mice at 24 months of age to young adult mice at 6 months of age – corresponding to ~30 years of age in humans (Figure 1A).

E-cadherin immunostaining of the epithelial layer in normal adjacent prostate to BPH and BPH glands was uniformly distributed and most intense along the cell membrane, with decreased staining observed in BPH glands as evidenced by a reduction of clearly demarked E-cadherin positive cell borders in BPH glands (Figure 1B) [6]. This diffuse reduction in E-cadherin staining was not observed in mice with a homozygous deletion of the E-cadherin gene in luminal epithelial cells [13] (Figure 1C). While these *Cdh1*^-/-^ mice contain many prostate epithelial cells lacking E-cadherin staining, they are often found surrounded by E-cadherin positive luminal epithelial cells in a mosaic pattern [13] (Figure 1C). This is likely the result of replenishment of luminal epithelial cells from a basal progenitor that was not ablated of the E-cadherin gene [19]. In order to more closely recapitulate the uniform reduction in E-cadherin expression observed in BPH glands of patients aged 50–77 years [6], we generated a cohort of mice with heterozygous deletion of E-cadherin and examined them at age 24 months. In this way, we could determine the impact of reduced E-cadherin expression (i.e., generated by heterozygosity in the mouse model) on the aged prostate and bladder. Similar to the pattern of E-cadherin immunostaining observed in prostate specimens from BPH patients, mice at 24 months of age displayed cell membrane E-cadherin expression in the epithelial layer of both Control and *Cdh1*^+/-^ mice, with a diffuse decreased expression in prostate glands of *Cdh1*^+/-^ mice in all lobes of the prostate (blue arrows, Figure 1D, center panel, Supplementary Figure 1). *Cdh1*^+/-^ mice also displayed glands with the mosaic pattern of E-cadherin negative cells surrounded by E-cadherin positive cells (black arrows, Figure 1D, right panel), similar to the pattern observed in *Cdh1*^-/-^ mice at 6 months of age [13].

### Impact of aging and E-cadherin deficiency on the murine prostate

Virgin male mice were generated with the following genotype combinations: PSA-Cre^+^; *Cdh1^flox/+^* (referred to herein as *Cdh1*^+/-^) and PSA-Cre^-^; *Cdh1^flox/+^* (Control). As an additional control for the possible effects of PSA-Cre, we also generated a cohort of three PSA-Cre^+^; *Cdh1*^+/+^ (PSA-Cre^+^) mice for histologic analyses. At 24 months of age, no statistically significant differences were observed in mean body, organ, or prostate mass between Control and *Cdh1*^+/-^ mice (Table 1). The prostates from PSA-Cre^+^ mice also appeared histologically normal and expressed E-cadherin in a pattern similar to PSA-Cre^-^; *Cdh1^flox/+^* controls (Supplementary Figure 2). There were no histologic defects detected in the kidney, spleen or testis and no difference in morbidity or mortality between groups at 24 months of age (data not shown). These findings suggest that the presence of PSA-Cre did not induce systemic defects.

**Table 1:** Mean body and organ mass of male mice at 24 months of age.

	**Control**	***Cdh1* ^+/-^**	***p*-value**
Body Mass (g)	39.8 ± 7.18	37.13 ± 5.03	0.647
Length (cm)	9.6 ± 0.34	9.7 ± 0.37	0.607
Ventral prostate (mg)	15.35 ± 6.5	16.17 ± 6.8	0.814
Dorsal-lateral prostate (mg)	32.1 ± 6.8	32.4 ± 15	0.952
Anterior prostate (mg)	55.8 ± 15.8	75.3 ± 41.3	0.211
Heart (mg)	217.4 ± 34	200.8 ± 33	0.425
Kidney (mg)	252.1 ± 22	257.6 ± 51	0.837
Spleen (mg)	114.9 ± 117	113.2 ± 61	0.484
Testis (mg)	148.0 ± 13.8	148.6 ± 15.2	0.480

Data represent mean ± Standard Deviation.

At 6 months of age, the prostates of control and *Cdh1*^+/-^ mice appeared histologically normal and did not display any epithelial pathologic changes (Table 2). Aging in control mice induced a nonsignificant increase in prostate hyperplasia and mPIN (murine prostatic intraepithelial neoplasia). The prostates of *Cdh1*^+/-^ mice at 24 months of age displayed a statistically significant increase in epithelial changes including epithelial hyperplasia and mPIN lesions compared to both age-matched controls and *Cdh1*^+/-^ mice at 6 months of age (Table 2, Supplementary Figure 3). One *Cdh1*^+/-^ mouse at 24 months of age (1/7 *Cdh1*^+/-^ vs. 0/9 *Cdh1*^+/+^, *p* = 0.44) developed prostate cancer.

**Table 2:** Impact of age and E-cadherin deficiency on prostatic epithelial pathologic changes in male mice.

	**None**	**Hyperplasia**	**mPIN**	**Carcinoma**
**Control**				
6 months of age				
Ventral lobe	7/7 (100%)	0/7 (0%)	0/7 (0%)	0/7 (0%)
Lateral lobe	7/7 (100%)	0/7 (0%)	0/7 (0%)	0/7 (0%)
Dorsal lobe	7/7 (100%)	0/7 (0%)	0/7 (0%)	0/7 (0%)
Anterior lobe	7/7 (100%)	0/7 (0%)	0/7 (0%)	0/7 (0%)
**Control**				
24 months of age				
Ventral lobe	3/9 (33%)	4/9 (44%)	4/9 (44%)	0/9 (0%)
Lateral lobe	3/9 (33%)	3/9 (33%)	3/9 (33%)	0/9 (0%)
Dorsal lobe	5/9 (56%)	3/9 (33%)	3/9 (33%)	0/9 (0%)
Anterior lobe	6/9 (67%)	2/9 (22%)	0/9 (0%)	0/9 (0%)
***Cdh1* ^+/-^**				
6 months of age				
Ventral lobe	3/3 (100%)	0/3 (0%)	0/3 (0%)	0/3 (0%)
Lateral lobe	3/3 (100%)	0/3 (0%)	0/3 (0%)	0/3 (0%)
Dorsal lobe	3/3 (100%)	0/3 (0%)	0/3 (0%)	0/3 (0%)
Anterior lobe	3/3 (100%)	0/3 (0%)	0/3 (0%)	0/3 (0%)
24 months of age				
Ventral lobe	0/7 (0%)	7/7 (100%)^**##^	6/7 (86%)^#^	0/7 (0%)
Lateral lobe	0/7 (0%)	7/7 (100%)^*##^	7/7 (100%)^*##^	1/7 (14%)
Dorsal lobe	0/7 (0%)^*^	7/7 (100%)^**##^	7/7 (100%)^*##^	1/7 (14%)
Anterior lobe	0/7 (0%)	7/7 (100%)^**##^	4/7 (57%)^*^	0/7 (0%)

Abbreviation: mPIN: murine prostatic intraepithelial neoplasia. Control vs. *Cdh1*^+/-^ at 24 months of age, ^*^p < 0.05, ^**^p < 0.01, *Cdh1*^+/-^ at 6 months of age vs. *Cdh1*^+/-^ at 24 months of age, ^#^*p* < 0.05, ^##^*p* < 0.01.

**Figure 2:**
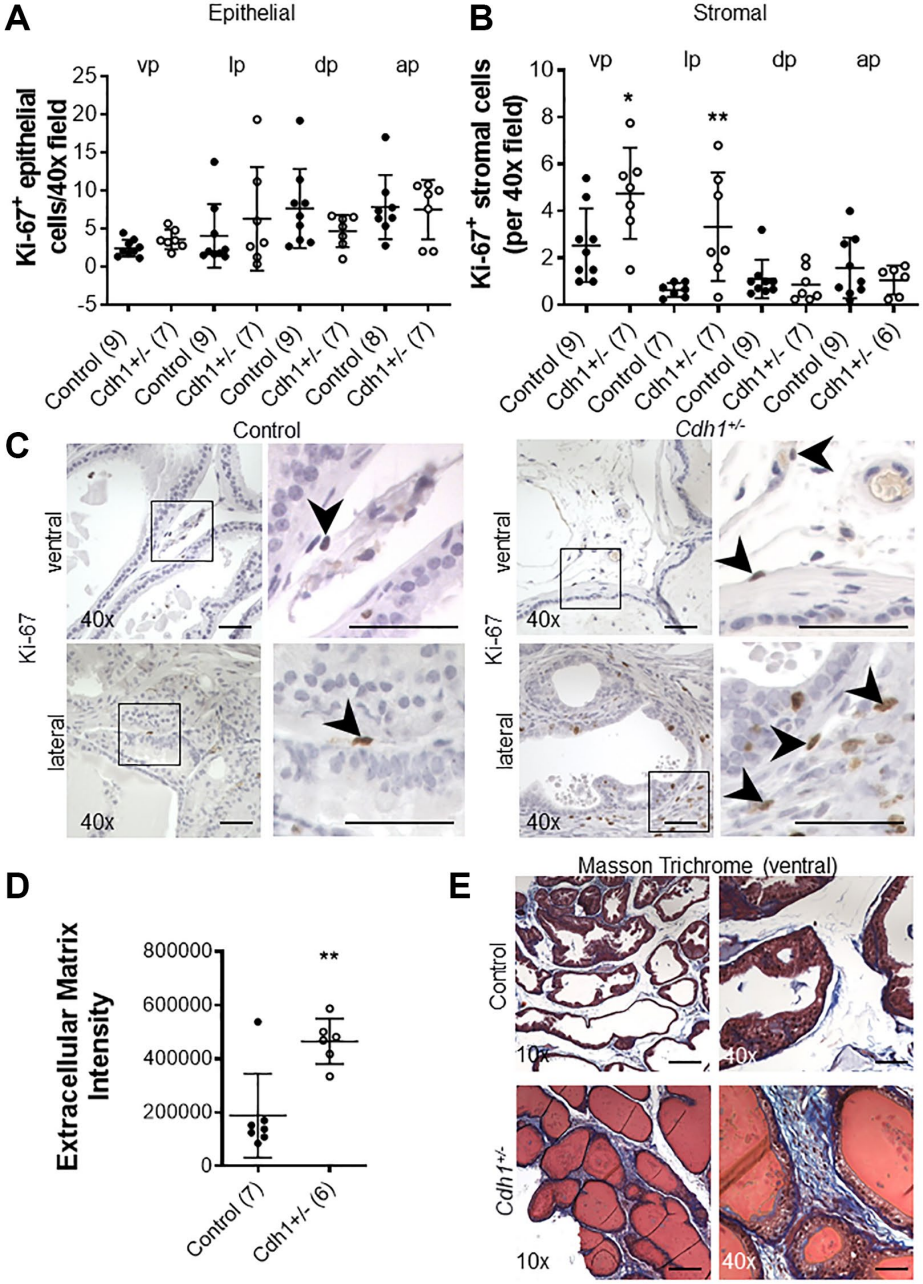
**Impact of prostate epithelial specific heterozygous E-cadherin loss on proliferation and extracellular matrix deposition in the murine prostate at 24 months of age.** (**A**) Quantification of Ki-67^+^ epithelial, and (**B**) stromal cells in the lobes of the prostate from Control and *Cdh1*^+/-^ mice at 24 months of age. Abbreviations: vp: ventral prostate; lp: lateral prostate; dp: dorsal prostate; ap: anterior prostate. (**C**) Ki-67 immunostaining (brown) in the stromal compartments of prostate ventral and lateral lobes. Black arrows indicate Ki-67^+^ cells in the stromal compartment. Original magnification, 40×, inset 40×. (**D**) Quantification of Masson’s trichrome staining of extracellular matrix (blue) in the stroma surrounding prostate glands from ventral prostate of Control and *Cdh1*^+/-^ mice at 24 months of age. Seven fields from each section were analyzed and an average score was determined for each mouse. (**E**) Masson’s trichrome staining in transverse sections of prostate ventral lobes in Control (top panels) and *Cdh1*^+/-^ mice (bottom panels). Original magnification, 10×, inset 40×. Data represent mean ± S.D, number of mice in each group in parentheses. Lobes which had been washed away during staining process were not quantified. ^*^p < 0.05, ^**^p < 0.01. (**D**) Scale bars indicate 200 μm in 10×, 50 μm 40×.

**Figure 3:**
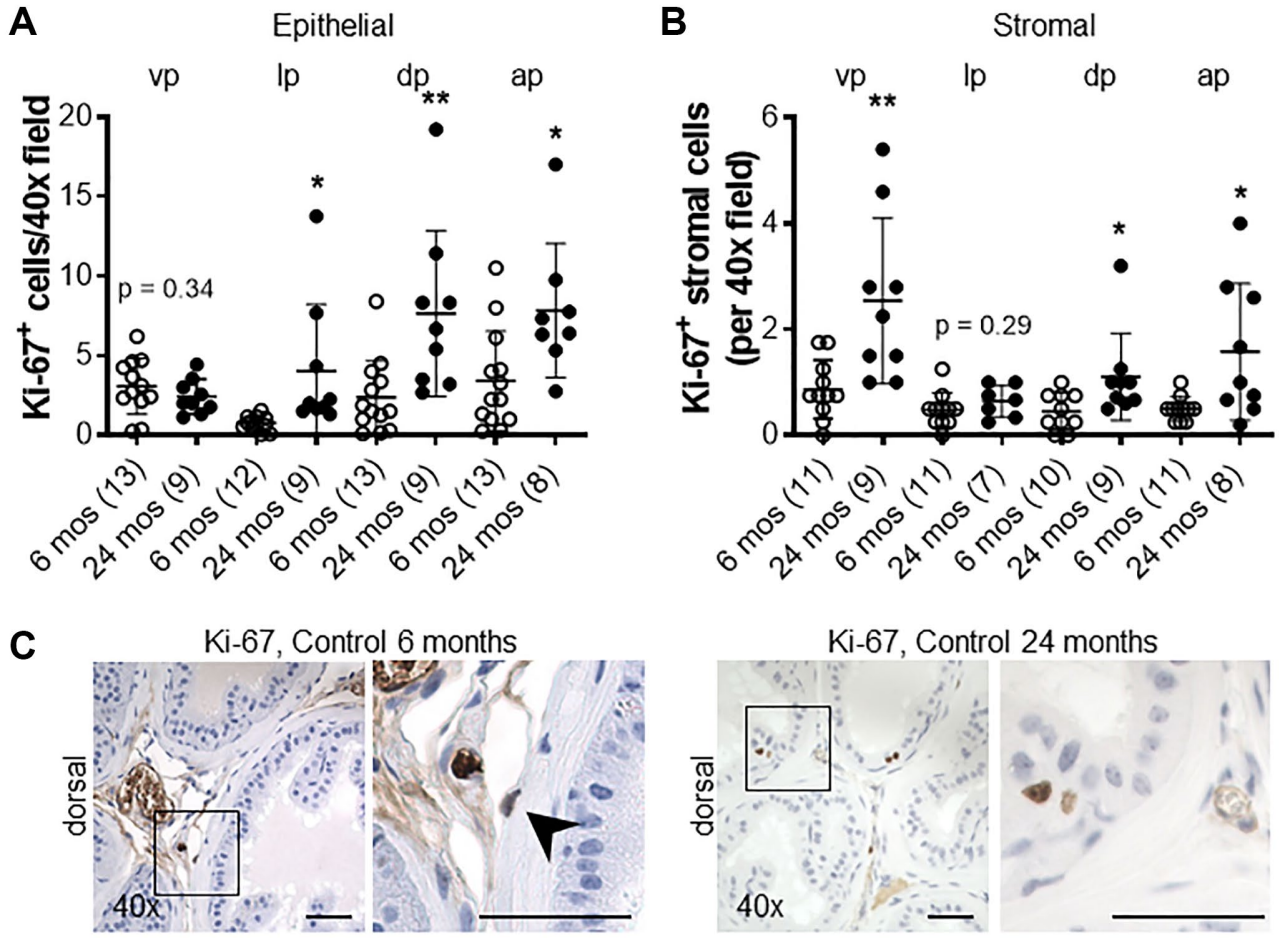
**Impact of aging on proliferation in the murine prostate.** (**A**) Quantification of Ki-67^+^ epithelial, and (**B**) stromal cells in the lobes of the prostate from control mice at 6 and 24 months of age. Abbreviations: vp: ventral prostate; lp: lateral prostate; dp: dorsal prostate; ap: anterior prostate. (**C**) Ki-67 immunostaining (brown) in dorsal lobes from control mice at 6 and 24 months of age. Black arrow indicates Ki-67^+^ stromal cell in control mice at 6 months of age. Original magnification, 40×, inset 40×. Data represent mean ± S.D, number of mice in each group in parentheses. Lobes which had been washed away during staining process were not quantified. ^*^p < 0.05, ^**^p < 0.01. Scale bars indicate 50 μm in 40×.

The proliferative marker Ki-67 was used to detect dividing cells in the prostates of *Cdh1*^+/-^ and control mice. There was no significant difference in the number of Ki-67-positive epithelial cells in all lobes of the *Cdh1*^+/-^ mice compared to controls (Figure 2A). In contrast, there was a statistically significant increase in Ki-67-positive stromal cells in the ventral and lateral lobes of *Cdh1*^+/-^ mice, indicative of stromal hyperplasia (Figure 2B, 2C). Masson’s trichrome staining showed an increase in extracellular matrix (ECM) deposition in the ventral lobes of *Cdh1*^+/-^ mice compared to control prostates (Figure 2D, 2E). In a post hoc comparison of control mice at 24 months of age to mice at 6 months of age, there was a significant increase in both epithelial and stromal proliferation in aged mice (Figure 3). We did not observe a significant increase in ECM deposition in aged mice compared to 6 months old controls (Supplementary Figure 4). These results suggest that aging is associated with an increase in epithelial and stromal proliferation, and that E-cadherin deficiency further increased stromal proliferation in the aged murine prostate.

**Figure 4:**
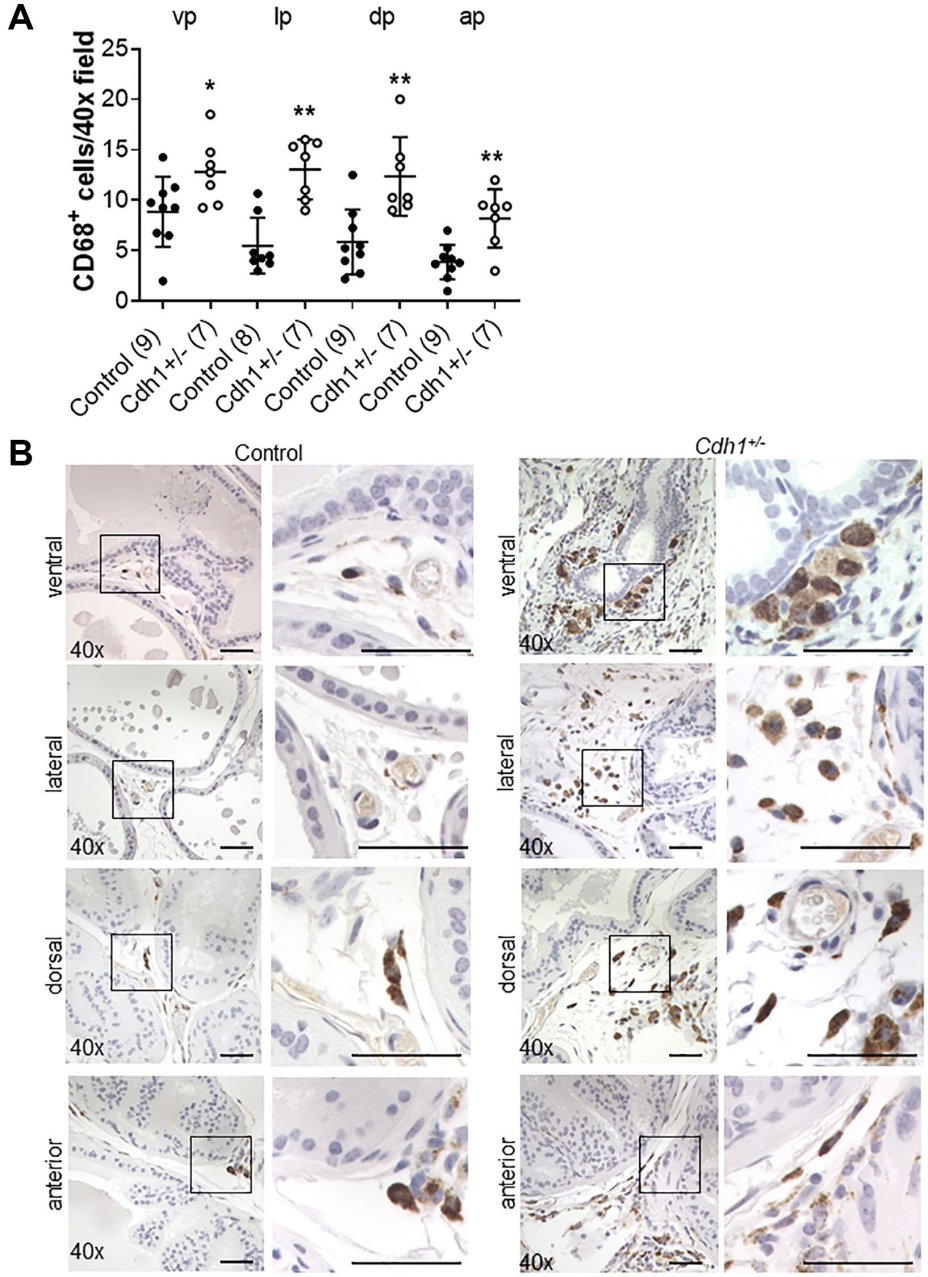
**Impact of E-cadherin-deficiency on macrophage density in the murine prostate at 24 months of age.** (**A**) Quantification of CD68^+^ macrophages in the prostate from Control and *Cdh1*^+/-^ mice at 24 months of age. Abbreviations: vp: ventral prostate; lp: lateral prostate; dp: dorsal prostate; ap: anterior prostate. Data represent mean ± S.D, number of mice in each group in parentheses. Lobes which had been washed away during staining process were not quantified. (**B**) CD68 immunostaining (brown) in transverse sections of prostate ventral, lateral, dorsal, and anterior lobes from Control and *Cdh1*^+/-^ mice at 24 months of age. Lobes which had been washed away during staining process were not quantified. Original magnification, 40×, inset 40×. ^*^p < 0.05, ^**^p < 0.01. Scale bars indicate 50 μm in 40×.

**Figure 5:**
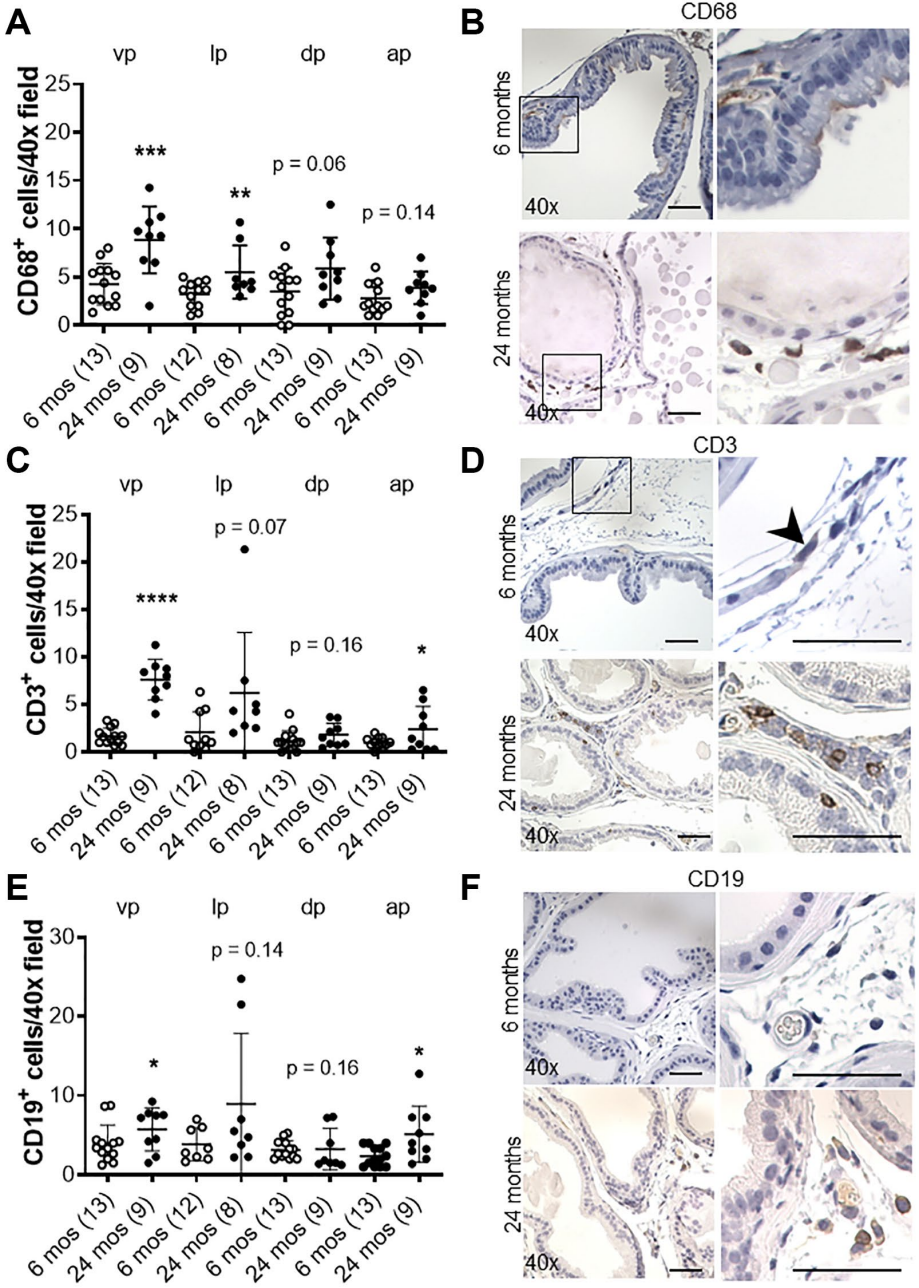
**Effects of age on inflammation in the murine prostate.** (**A**) Quantification of CD68^+^ macrophages in the prostate from control mice at 6 and 24 months of age. Abbreviations: vp: ventral prostate; lp: lateral prostate; dp: dorsal prostate; ap: anterior prostate. (**B**) CD68 immunostaining (brown) in transverse sections of prostate ventral lobes at 6 and 24 months of age. (**C**) Quantification of CD3^+^ T-cells in the prostate from control mice. (**D**) CD3 immunostaining (brown) in transverse sections of prostate ventral lobes. Black arrow indicates CD^+^ cell. (**E**) Quantification of CD19^+^ B-cells in the prostate from control mice. (**F**) CD19 immunostaining (brown) in transverse sections of prostate ventral lobes. Data represent mean ± S.D, number of mice in each group in parentheses. Lobes which had been washed away during staining process were not quantified. Original magnification, 40×, inset 40×. ^*^p < 0.05, ^**^p < 0.01. Scale bars indicate 50 μm in 40×.

We recently showed that in aging human prostate, increased inflammation is correlated with down-regulation of epithelial barrier proteins E-cadherin [6], which was previously found to be down-regulated in BPH [14–16]. Previous studies have also demonstrated an association between macrophage infiltration and fibrosis in the murine prostate [20]. Thus, we analyzed the number of CD19-positive B-cells, CD3-positive T-cells, and CD68-positive macrophages in aged mice. Lymphomas composed of both CD19-positive B cells and CD3-positive T-cells were observed and identified by a board-certified animal pathologist (LHR, V.M.D.) in the prostates of both controls (4/9) and *Cdh1*^+/-^ mice (3/7) at 24 months of age, while neither cohort displayed lymphoma at 6 months of age (0/7 in controls, 0/3 in *Cdh1*^+/-^ mice). Although the age-related increased incidence was not statistically significant (*p* = 0.07 in controls, *p* = 0.48 in *Cdh1*^+/-^ mice), the spontaneous development of lymphoma in aged C57BL/6J mice is common [21]. The incidence of lymphoma was similar in both cohorts (*p* = 1.00), and this made it difficult to detect an increase in B-cells or T-cells in the prostates of *Cdh1*^+/-^ mice compared to age-matched controls at 24 months of age. However, there was a significant increase in the number of CD68-positive macrophages in all lobes of the prostates of *Cdh1*^+/-^ mice at 24 months of age (Figure 4), and a significant increase in macrophages, T-cells, and B-cells in the prostates of control mice at 24 months of age compared to controls at 6 months of age (Figure 5). These results suggest that heterozygous deletion of E-cadherin in aged animals was associated with a further increased number of macrophages in the prostate of aged animals.

### Voiding behavior and conscious cystometry in Cdh1^+/-^ mice at 24 months of age

Previously, we and others have reported an increased urinary frequency [22] and bladder overactivity in mice and rat models of bacterial- or chemical-induced prostatic inflammation [23, 24]. We also reported that mice with homozygous deletion of *Cdh1* developed prostatic inflammation and displayed an increase in the number of voids, non-voiding contractions and a decrease in the average single voided volume compared to controls at 6 months of age [13]. Post-hoc comparison of control mice at 6 months of age vs. 24 months of age revealed that aged mice displayed a significant decreased average single voided volume in voiding assays during the four-hour observation period despite larger bladder mass at necropsy and a trend increase of ICI during cystometry in 24 months-old control mice (Table 3). Because bladder voiding efficiency calculated as a percent ratio of voided volume against bladder capacity was lowered to 66% in aged control mice compared to control mice at 6 months of age (Table 3), it is assumed that aged mice exhibited an underactive bladder condition resulting in inefficient voiding, in line with a previous study in which voiding dysfunction due to reduced bladder contractility was observed with aging in male mice [12]. In addition, there was a trend decrease in total urine output volume (1101.2 vs. 567.7, *p* = 0.07) during four hours in control mice at 24 months of age compared to control mice at 6 months of age (Table 3).

**Table 3:** Impact of aging and E-cadherin deficiency on bladder voiding characteristics and void spot assays in male mice.

	**6 months Control (*n* = 6)**	**24 months Control (*n* = 6)**	***p*-value 6 months vs. 24 months Control**	**24 months *Cdh1*^+/-^ (*n* = 5)**	***p*-value 24 months Control vs. *Cdh1*^+/-^**
**Voiding spot assay**					
Number of voids	1.80 ± 0.837	2.50 ± 1.64	0.41	2.00 ± 1.41	0.61
Average single voided volume (μl)	608.8 ± 103.7	217.8 ± 155.1	0.001	278.2 ± 243.2	0.64
Total urine/4 hr (μl)	1101.2 ± 533.7	567.7 ± 348.2	0.07	625.8 ± 473.8	0.82
Bladder volume (cm^3^)^*^	NA	0.931 ± 0.99	NA	3.31 ± 3.46	0.07
Bladder mass (mg)^*^	304 ± 152	567 ± 69	0.0001	422 ± 376	0.44
**Awake cystometry**					
Number of non-voiding contractions (NVC/min)^#^	0.210 ± 0.217	0.098 ± 0.071	0.261	0.364 ± 0.176	0.008
Intercontraction interval (ICI/sec)^#^	182.98 ± 82.86	317.3 ± 62.2	0.010	506.8 ± 239.6	0.090
Bladder capacity (μl)^#^	NA	92.6 ± 42.6	NA	157.7 ± 47.7	0.053
Bladder voiding efficiency (%)^#^	NA	66.3 ± 20.1	NA	56.0 ± 22.3	0.478

Data represent mean ± Standard Deviation. Number of voids, average single voided volume, and total urine measured over four-hour period of observation for void spot assay. ^*^Bladder mass and volume determined at necropsy. ^#^NVC, ICI, bladder capacity and bladder voiding efficiency determined by awake cystometry. Abbreviations: NA: not available; NVC: non-voiding contractions; ICI: intercontraction interval.

**Figure 6:**
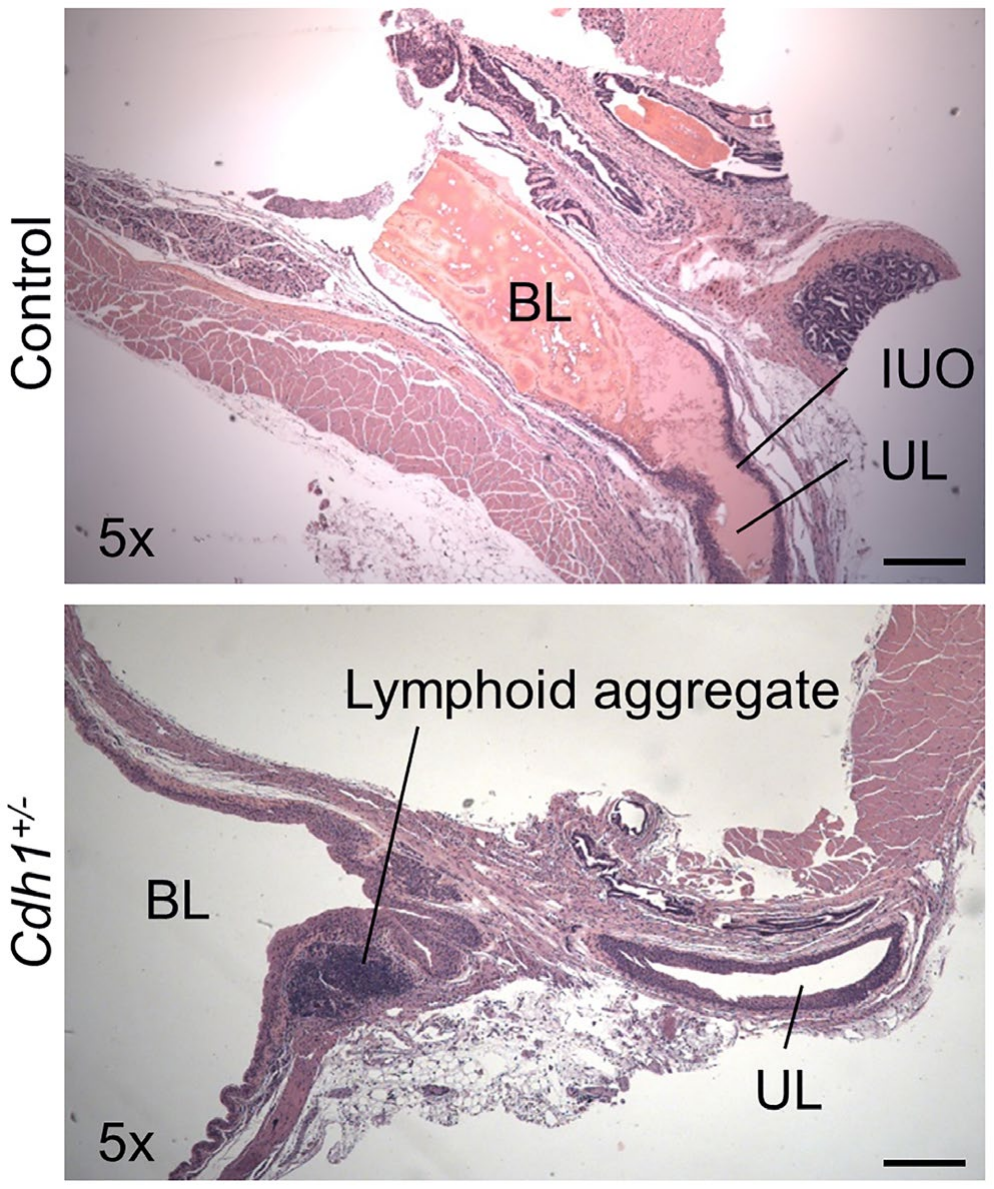
**Impact of prostate-specific E-cadherin deficiency on bladder histology in mice analyzed at 24 months of age.** H&E staining of transverse sections of bladder and bladder neck from Control and *Cdh1*^+/-^ mice at 24 months of age. Abbreviations: BL: bladder lumen; UL: urethra lumen; IUO: Internal urethral orifice. Lymphoid aggregates (shown in *Cdh1*^+/-^ image) were observed in both control and *Cdh1*^+/-^ mice. Original magnification, 5×, scale bars indicate 400 μm in 5×.

**Figure 7:**
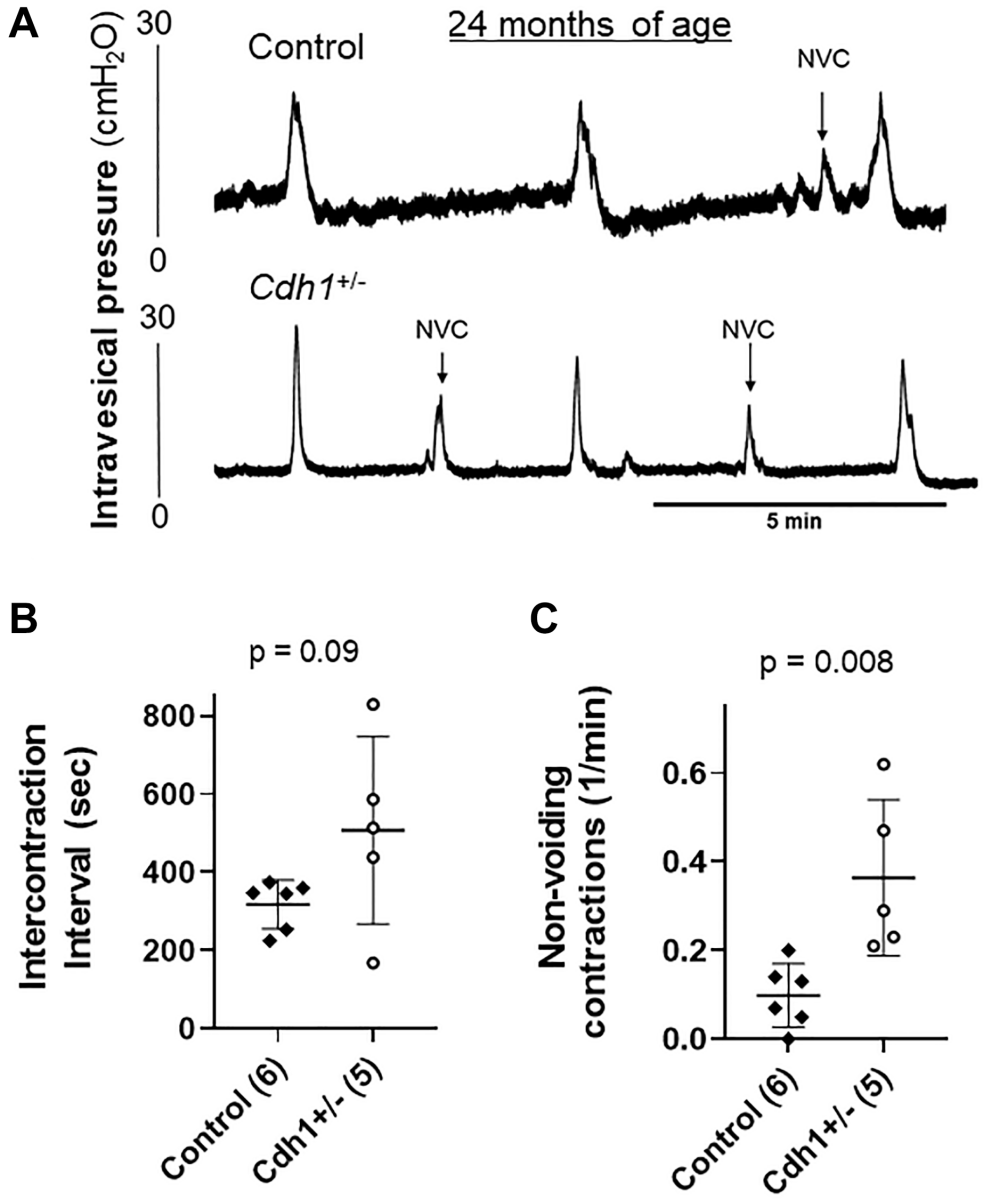
**Impact of prostate-specific E-cadherin deficiency on bladder cystometry in mice analyzed at 24 months of age.** (**A**) Representative cystometric tracings for Control and *Cdh1*^+/-^ mice at 24 months of age. (**B**) Intercontraction interval. (**C**) Number of non-voiding contractions (NVC). Number of mice in each group in parentheses.

Although the difference was not statistically significant, the measured bladder volume at necropsy of *Cdh1*^+/-^ mice at 24 months of age was much larger than that of age-matched control mice (3.31 vs. 0.931 cm^3^, *p* = 0.068) (Table 3). In accordance, aged *Cdh1*^+/-^ mice displayed trend increases in ICI (*p* = 0.090) and bladder capacity (*p* = 0.053) in cystometry compared to age-matched controls (Table 3) although enlarged bladders did not display obvious histological defects compared to age-matched controls (Figure 6). However, because bladder voiding efficiency was not different between aged *Cdh1*^+/-^ mice and age-matched control mice (56 vs. 66%, *p* = 0.478) (Table 3), the aging-related underactive bladder condition does not seem to be significantly worsened by E-cadherin downregulation. In contrast, cystometric tracing of awake *Cdh1*^+/-^ mice demonstrated a significantly increased mean number of non-voiding contractions (NVC) per minute compared to controls (Figure 7) in aged mice, suggesting that prostatic defects induced by E-cadherin heterozygosity contributed to the development of bladder overactivity during the storage phase in aged mice. Cystometric analysis was not performed on *Cdh1*^+/-^ mice at 6 months of age since they did not display any prostatic defects. Taken together, these changes suggest that aging contributes to bladder underactivity during the voiding phase function while decreased E-cadherin in the aging prostate can additionally induce bladder overactivity during the storage phase in aged mice.

## DISCUSSION

We previously demonstrated that homozygous *Cdh1* knockout in prostate luminal epithelial cells induced prostatic inflammation and hyperplasia and subsequent bladder overactivity in mice at 6 months of age [13]. Thus, E-cadherin plays an important role in maintenance of adult prostate and prostate inflammation induced by E-cadherin deficiency can cause alterations in bladder function. However, in benign prostatic conditions (i.e., BPH), E-cadherin is reduced but not completely ablated. Here, we sought to determine whether partial loss of E-cadherin rather than complete ablation could also contribute to the development of a BPH/LUTS-like phenotype, particularly in aged animals given the association of BPH/LUTS development with aging in humans.

At 6 months of age, neither control nor *Cdh1*^+/-^ mice displayed any histologic defects in the prostate, but mice in both groups developed a significant increase in hyperplasia and mPIN lesions at 24 months of age, suggesting that aging contributes to prostate epithelial proliferation. *Cdh1*^+/-^ mice displayed an increase in hyperplastic and neoplastic lesions compared to age-matched controls (Table 2). The increased incidence of both hyperplastic and neoplastic lesions in aged *Cdh1*^+/-^ mice is not surprising. Both BPH/LUTS and prostate cancer are strongly associated with age and with decreased E-cadherin expression, however they are two distinct diseases [6, 14, 16, 25, 26]. Although human BPH is not thought to become prostate cancer and is not associated with an increase in prostate cancer risk, over 80% of prostate cancer patients also have histological BPH [27]. Furthermore, prostate cancer, at least in early stages, is not associated with LUTS [28]. Thus, it may not be surprising that we observe age-dependent effects in *Cdh1*^+/-^ mice that extend beyond benign alterations, specifically, an enhancement in the incidence rate of mPIN (44% in control vs. 100% in *Cdh1*^+/-^, *p* = 0.0337 in ventral prostate). These results suggest that prostate cancer in aged men may not only be associated with complete loss of E-cadherin expression [26], but also be promoted by even subtle changes in cellular function requiring optimal functioning of E-cadherin.

The development of LUTS in BPH patients is associated with elevated prostate inflammation [25]. At 6 months of age, homozygous deletion of E-cadherin in the murine prostate induced an increased number of non-voiding contractions, an increased number of voids and a smaller average voiding volume without changes in total urine volume [13]. However, bladder dysfunction in mice may not require a complete ablation of E-cadherin and be driven by aging. Aged control mice also displayed increased prostate inflammation (Figure 5) and reduced voided volume compared to controls at 6 months of age (Table 3). The average bladder mass of aged control mice was about twice that of controls at 6 months of age; and although the differences were not statistically significant, an associated trend increase in bladder capacity (i.e., increased ICI) with small voided-volume per micturition and reduced bladder voiding efficiency in aged compared to young animals suggest that aging mice develop an underactive bladder condition (Table 3). In aged *Cdh1*^+/-^ mice, the average bladder volume was about three times that of age-matched controls at necropsy, and bladder capacity in cystometry tended to be increased (Figure 7, Table 3). However, bladder voiding efficiency was not significantly different between aged *Cdh1*^+/-^ mice and age-matched controls (Table 3), suggesting that the aging-related underactive bladder condition during the voiding phase was not significantly worsened by E-cadherin heterozygosity. In contrast, aged *Cdh1*^+/-^ mice displayed a significant increase in the number of non-voiding contractions during the storage phase (Figure 7, Table 3), indicative of an overactive bladder condition. Taken together, these findings suggest that E-cadherin deficiency associated with increased prostatic inflammation in the aging prostate can contribute to the development of bladder overactivity as evidenced by increased NVC during the storage phase in aged mice, possibly due in part to prostatic macrophage infiltration and fibrosis.

Chronic low-grade systemic inflammation is a hallmark of aging (termed inflammaging) [1] and plays a role in the pathogenesis of several age-related chronic diseases [2]. Accumulating effects from redox stress, mitochondrial damage, senescence, epigenetic modifications, inflammatory disease, and frailty likely all play a role in promoting inflammaging (Reviewed in [3]). BPH is a prevalent prostate disease that has been strongly correlated with increased age and is frequently associated with inflammation [4]. Aging contributes to an increase in prostatic inflammation, and is a significant risk factor for the development and progression of BPH/LUTS [29]. It has been shown that aged mice in both sexes similarly develop characteristics of voiding dysfunction with low bladder voiding efficiency due to reduced bladder muscle contractility, which mimics the condition of underactive bladder often observed in aged humans [12]. Furthermore, although cell proliferation in the rodent prostate cannot directly compress the urethra due to a lack of capsular structure enclosing the prostate, unlike humans, aged male mice reportedly develop bladder outlet obstruction due to increased collagen deposition and glandular hyperplasia within the prostatic urethra [11, 30]. Thus, both bladder underactivity and partial urethral obstruction are likely to contribute to voiding dysfunction detected in aged male mice with or without E-cadherin heterozygosity in this study. In addition, we recently showed that decreased E-cadherin in the human prostate was associated with aging and was correlated with increased inflammation [6], and we and others have shown that E-cadherin is decreased in BPH [14–16]. This study demonstrated that E-cadherin downregulation with enhanced prostatic inflammation further promoted BPH-like phenotypic changes in the prostate and bladder overactivity, which is one of the hallmark manifestations of male LUTS associated with BPH, in addition to voiding dysfunction, to which the aging process seems to be an important contributing factor. Taken together, our findings here demonstrate that aging promotes the prostate inflammation and changes in bladder function in *Cdh1*^+/-^ mice which are characteristic of age-related diseases such as BPH/LUTS.

Overall, these results demonstrate that age-dependent prostatic defects specific to *Cdh1*^+/-^ mice include increased macrophage recruitment, an increased incidence in prostate stromal hyperplasia and extracellular matrix deposition as well as bladder overactivity. Aging contributes to an increase in prostate inflammation evident as increased presence of T-cell, B-cell, and macrophages, and declines in voiding function evident as large bladder capacity and decreased voided volume per micturition with low bladder voiding efficiency. The presence of macrophages in the prostate can be further exacerbated by E-cadherin loss potentially contributing to an increase in proliferation and stromal fibrosis, additionally inducing bladder overactivity evident as increased NVCs in aged mice. However, urinary frequency in aged mice was not significantly affected by E-cadherin heterozygosity, possibly due to an aging-induced reduction in bladder sensation. Heterozygous E-cadherin deficiency in the prostate of aged mice induced a milder phenotype than that previously reported mice with homozygous E-cadherin deletion [13], providing a tool to study the impact of more mild to moderate, chronic E-cadherin deficiency in aged mice. This slow progressing, age-dependent phenotype induced by prostate-specific heterozygous E-cadherin loss recapitulates key characteristics observed in human BPH and LUTS and is distinct from other rodent models of BPH/LUTS. This model will be a useful resource for exploring whether targeting prostate inflammation or more specifically, macrophage inflammation or fibrosis, via pharmacologic or lifestyle interventions can delay or reduce the development or progression of symptomatic BPH/LUTS.

## MATERIALS AND METHODS

### E-cadherin immunostaining in human prostate specimens

E-cadherin immunostaining was performed on sections from a previously described experimental cohort of prostate tissue specimens [6, 31], which included 14 patients who received simple prostatectomy for symptomatic BPH, and six young healthy organ donors without any history of chemo-, radio-, or hormone therapy [31]. BPH specimens were composed of mixed hyperplastic nodules with both glandular and stromal expansion. The “deidentified” specimens were retrieved from the clinical files of UPMC by the University of Pittsburgh Biospecimen Core (PBC) with approval from the University of Pittsburgh Institutional Review Board for this research project under protocol #17010177. PBC also provided deidentified pathology reports for the patients constituting the study cohort through their IRB-approved Honest Broker System, HB015, to ensure research integrity. All participating patients or their next of kin provided informed consent for the banking protocol.

Samples were fixed in 10% formalin for at least 24 hours, then embedded in paraffin, sectioned at 5 μm. Immunohistochemical staining was performed on five-micron sections of paraffin-embedded prostate tissue specimens. Briefly, sections were deparaffinized and rehydrated through a graded series of ethanol. Heat-induced epitope retrieval was performed using a BioCare Decloaking Chamber (BioCare Medical, Pacheco, CA, USA), followed by 5 minutes rinsing in TBS buffer. Sections were stained with rabbit monoclonal anti-E-cadherin (ab76319, EP913(2)Y, Abcam, Cambridge, MA, USA), then counterstained in hematoxylin and cover-slipped. Stained sections were imaged with a Leica DM LB microscope (Leica Microsystems Inc., Bannockburn, IL, USA) equipped with an Imaging Source NII 770 camera (The Imaging Source Europe GmbH, Bremen, Germany) and NIS-Elements Documentation v 4.6 software (Nikon Instruments, Inc., Mellville, NY, USA). All tissues were examined by a board-certified genitourinary pathologist (R.D.) using light microscopy.

### Mice

All animal studies were reviewed and approved by the Institutional Animal Care and Use Committee (IACUC) of the University of Pittsburgh and were conducted in strict accordance with the standards for humane animal care and use as set by the Animal Welfare Act and the National Institutes of Health guidelines for the use of laboratory animals under Animal Welfare Assurance number A3187-01. Animals were provided water and irradiated chow (Prolab^®^ IsoPro^®^ RMH 3000 5P75, LabDiet, St. Louis, MO, USA) *ad libitum*. Male mice were generated by cross-breeding the B6.129-*Cdh1^tm2Kem/J^* mouse [32] (JAX stock #005319, The Jackson Laboratory, Bar Harbor, ME, USA) with the inducible Cre line PSA-CreER^T2^ (generously provided by Dr. Pierre Chambon and Dr. Daniel Metzger, IGBMC, Illkirch, France) on a C57BL/6J background [33]. Experimental cohorts were virgin male mice that were either hemizygous or negative for the Cre allele (PSA-Cre^+^ or PSA-Cre^-^) and had heterozygous or wild-type expression of *Cdh1* floxed alleles (*Cdh1^flox/+^* and *Cdh1*^+/+^) resulting in genotype combinations: PSA-Cre^+^; *Cdh1^flox/+^*, PSA-Cre^-^; *Cdh1^flox/+^*, or PSA-Cre^+^; *Cdh1*^+/+^. Mice herein were referred to as *Cdh1*^+/-^, control and PSA-Cre+, respectively. Genotyping was performed using PCR analysis of mouse tail genomic DNA at age 21 days and muscle genomic DNA after euthanization as previously [13].

Tamoxifen induction of Cre activity in male mice containing the PSA-CreER^T2^ allele was performed as previously for 5 consecutive days at 7 weeks of age [13, 34]. Tamoxifen (Sigma Chemical Co., St. Louis, MO, USA) suspended in Kolliphor EL (Sigma) 3 mg/40 g of body weight was injected i.p. daily. All animals in the study were treated with the same regimens of tamoxifen injections and were examined at 6 months or 24 months of age. Body mass was measured using a triple beam balance immediately prior to euthanization. Length was determined by measuring the animal from the nose to the anus [35]. Bladder was measured *in situ* in three dimensions with a precision caliper as described [36] and bladder volume was estimated by calculating the volume of an ellipsoid in cubic millimeters [37]. The heart, spleen, left kidney, left testis and lower urinary tract were removed and fixed in 10% formalin. Fixed tissues were cleaned of excess fat and membrane with phosphate-buffered saline; mass of each organ and each prostate lobe was determined after blotting with filtration paper to remove excess liquid.

### Histopathologic analysis of murine specimens

Murine tissue samples were fixed in 10% formalin for at least 24 hours, then embedded in paraffin, sectioned at 5 μm, and stained with hematoxylin and eosin. All tissues were examined by a board-certified animal pathologist in a blinded fashion (LHR, V.M.D.). Prostatic defects were identified as epithelial hyperplasia, stromal hyperplasia, inflammation and mPIN and adenocarcinoma per the criteria published by Shappell, et al., commonly used to score prostate lesions in transgenic mouse models [38].

### Immunohistochemistry and Masson’s trichrome staining of murine specimens

Immunohistochemical stains were performed on five-micron sections of paraffin-embedded murine tissue specimens as described previously [39]. Briefly, sections were de-paraffinized and rehydrated through a graded series of ethanol. Heat-induced epitope retrieval was performed using a BioCare Decloaking Chamber (BioCare Medical, Pacheco, CA, USA), followed by 5 minutes rinsing in TBS buffer. Primary antibodies for immunostaining of murine tissue sections were mouse monoclonal anti-E-cadherin (Clone 36, #610181, BD Biosciences, San Jose, CA, USA), rabbit monoclonal anti-Ki-67 (D3B5, Cell Signaling Technology, Danvers, MA, USA), rabbit monoclonal anti-CD3 (P, CP215, BioCare Medical), rabbit monoclonal anti-CD3 (D4V8L, #99940, Cell Signaling Technology), CD19 (D4V4B, #90176, Cell Signaling Technology), and mouse monoclonal anti-CD68 (KP1, CM033, BioCare Medical). Slides were then counterstained in hematoxylin and coverslipped. Masson’s Trichrome histochemical staining was performed using HT15 (Sigma Aldrich, St. Louis, MO, USA) according to the manufacturer’s protocol. Stained sections were imaged with a Leica DM LB microscope (Leica Microsystems Inc., Bannockburn, IL, USA) equipped with an Imaging Source NII 770 camera (The Imaging Source Europe GmbH, Bremen, Germany) and NIS-Elements Documentation v 4.6 software (Nikon Instruments, Inc., Mellville, NY, USA). All tissues were examined by a board-certified veterinary pathologist (L.H.R.) and a board-certified genitourinary pathologist (R.D.) using light microscopy.

Mean Ki-67-positive cell density was determined by analysis of sections from at least six independent mice from each genotype and from all lobes of the prostate. The average number of Ki-67-positive luminal epithelial cells or stromal cells in each prostate lobe for each mouse was determined from at least four nonoverlapping fields imaged at 40× magnification. The average number of CD19-positive B cells, CD68-positive macrophages and CD3-positive T cells was also determined similarly for each prostate lobe in each mouse.

Extracellular matrix (ECM) was quantified by calculating the average intensity of blue staining in Masson’s trichrome stain using Adobe PhotoShop 2021, as described [40]. Seven fields from each section were analyzed and an average score was determined for each mouse.

### Void spot assay

Mice were placed in metabolic cages and voiding behavior was evaluated for 4 hours. Filter paper was placed under the wire mesh bottom of each metabolic cage and urine stains on the paper were analyzed to determine voiding behavior and voided volumes using the Voided Stain on Paper (VSOP) method [41]. Before each analysis, mice were treated with subcutaneous injections of sterile water (20 μl/g body weight) to enhance urine production. The voided volume for each urine spot was calculated using ImageJ (NIH, Bethesda, MD, USA).

### Cystometry

Mice were examined by cystometric investigation under a conscious condition as previously [23]. Under isoflurane anesthesia, following a lower midline abdominal incision, the bladder was exposed and catheterized with PE-50 tubing (Clay Adams Division of Becton Dickinson, Parsippany, NJ, USA) inserted through the bladder dome. After recovery from anesthesia, conscious mice were placed in a restraining cage (Economy holder 15 to 30 g, Kent Scientific, Torrington, CT, USA). After 1–2 hours of acclimation, saline was infused into the bladder at 0.01 ml/min to induce micturition. At least three reproducible micturition cycles were recorded after an initial stabilization period (60 min). After several micturition cycles, the saline infusion was stopped to evaluate intercontraction intervals (ICI), and non-voiding contractions (NVCs), bladder capacity and bladder voiding efficiency using Chart software (AD Instruments, Colorado Springs, CO, USA). NVCs were defined as rises in intravesical pressure that exceeded 20% of voiding pressure over the baseline without fluid elimination from the urethral orifice during bladder filling.

Post-hoc comparison of data from control mice at 24 months of age (PSA-Cre-; *Cdh1^flox/+^*) to previously generated control mice at 6 months of age (PSA-Cre-; *Cdh1^flox/flox^*) [13] was performed to determine whether prostate inflammation and bladder function were altered with aging. Control mice from both 6 months and 24 months of age cohorts were treated with tamoxifen at 7 weeks of age.

### Statistical analysis

Comparisons between groups were calculated using the student’s *t*-test or two-tailed Fisher exact test using the method of summing small *p*-values as appropriate. The Pearson correlation coefficient was used to determine the correlation between extracellular matrix density, proliferation, and infiltration of inflammatory cells in the combined samples from aged mouse prostate (control and *Cdh1*^+/-^). A *p*-value of *p* < 0.05 was considered significant. GraphPad Prism version 9 was used for graphics (GraphPad Software, San Diego, CA, USA). Values are expressed as mean ± S.D.

## Supplementary Materials

Supplementary Figures
