# Supplementary material for: E-cadherin deficiency promotes prostate macrophage inflammation and bladder overactivity in aged male mice
Source: Aging (Albany NY). 2022 Mar 31;14(7):2945–65. doi: 10.18632/aging.203994 (PMC9037276; doi:10.18632/aging.203994)
Supplement: Supplementary Figures [file aging-14-203994-s001.pdf]

Supplementary Figures

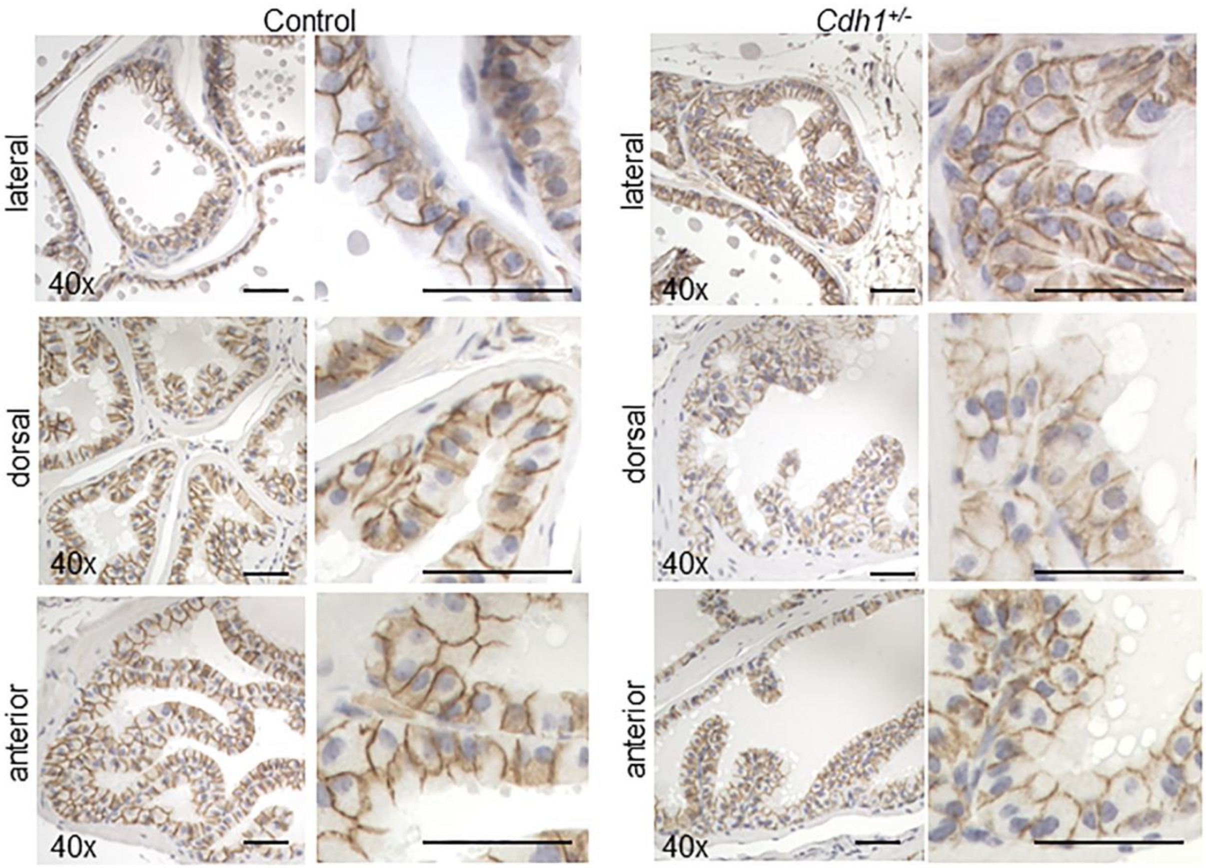

Supplementary Figure 1. Expression of E-cadherin in the prostate lateral, dorsal and anterior lobes of control and *Cdh1*$^{+/−}$ mice at 24 months of age. Original magnification, 40×, inset 40×. Scale bars indicate 50 µm in 40×.

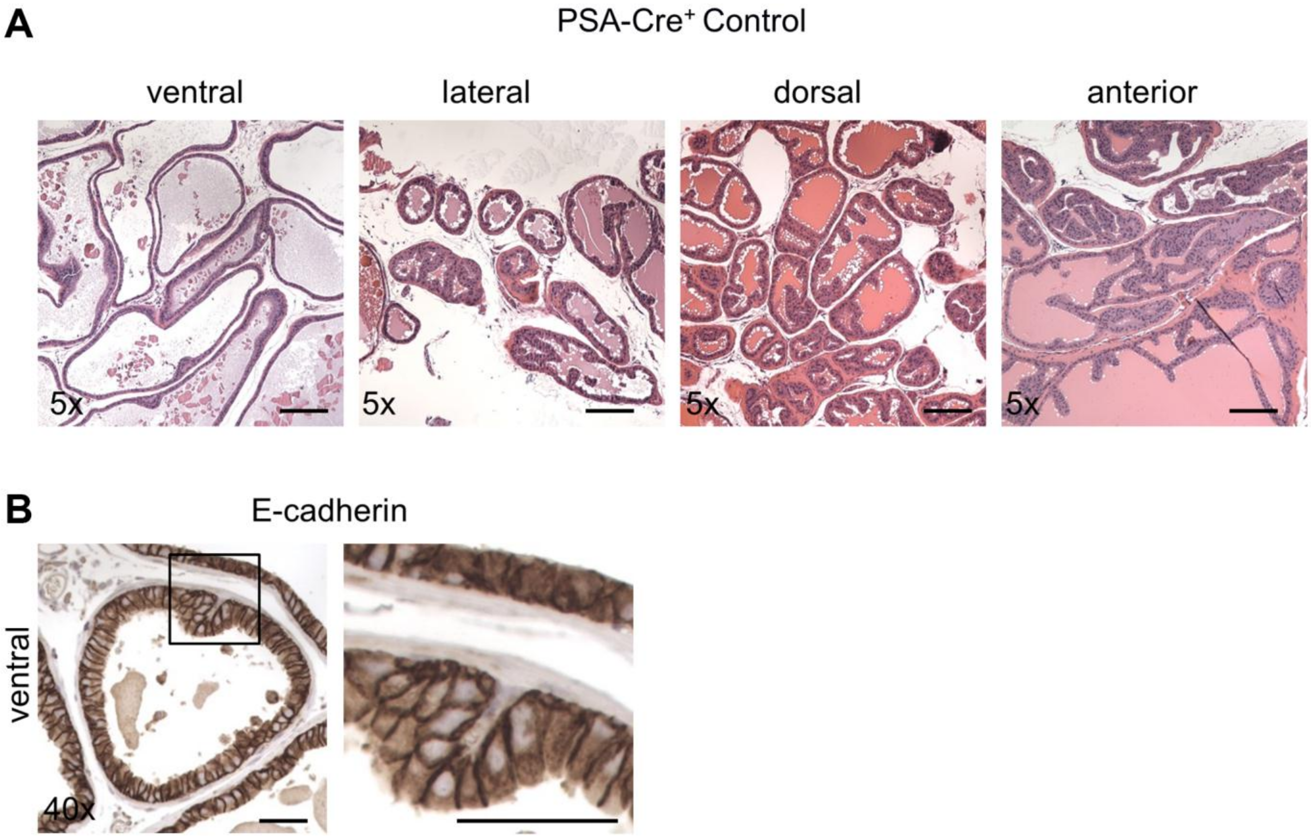

Supplementary Figure 2. PSA-Cre^{+} Control mouse prostate at 24 months of age. (**A**) H&E staining of transverse sections of prostate ventral, lateral, dorsal, and anterior lobes of PSA-Cre^{+} Control mice at 24 months of age. (**B**) E-cadherin immunostaining in prostate ventral lobe of PSA-Cre^{+} Control mice at 24 months of age. Original magnification, 5×, inset 40×. Scale bars indicate 50 µm in 40×.

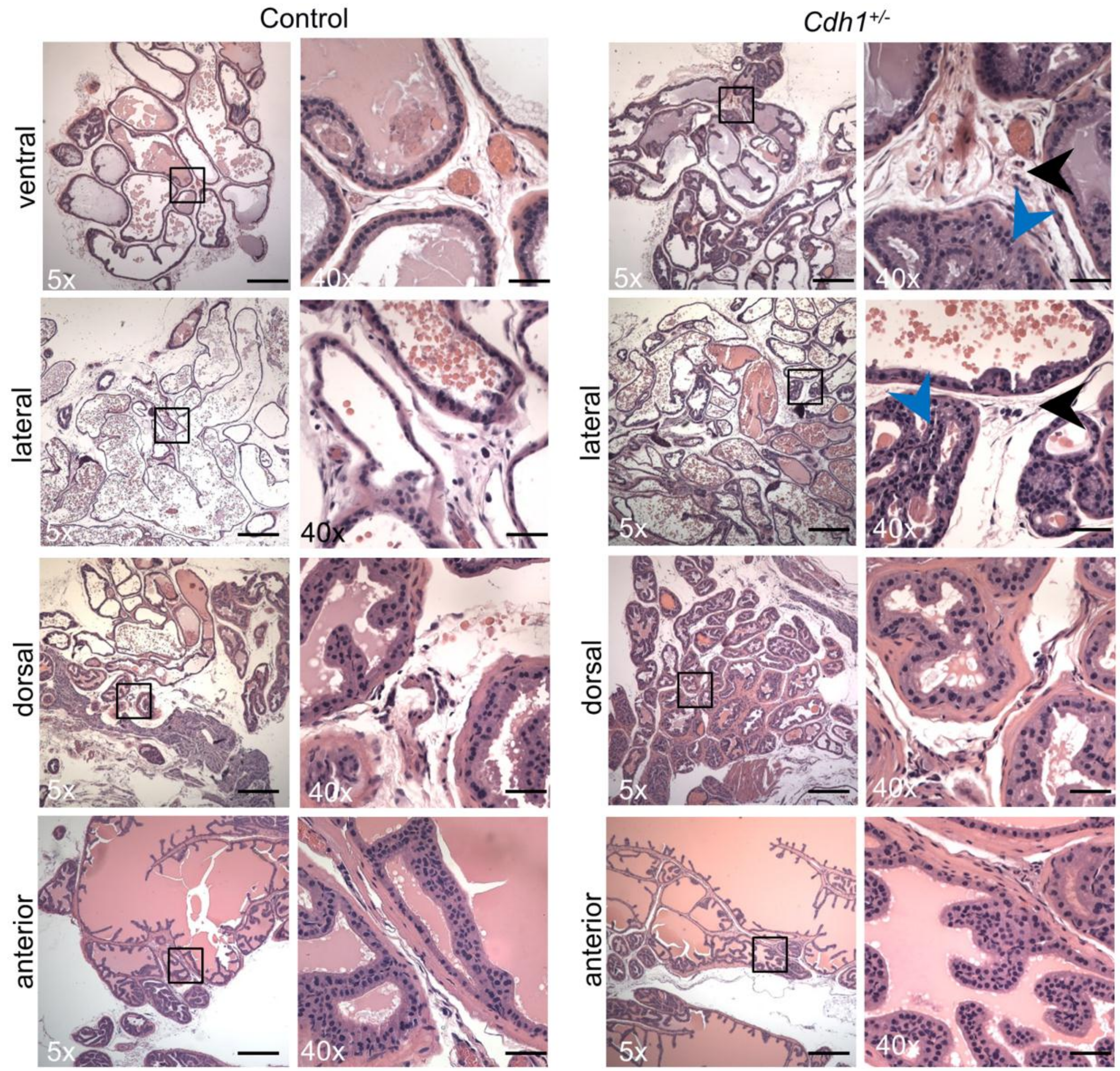

Supplementary Figure 3. Histology of control PSA-Cre⁻; *and Cdh1*$^{+/-}$ murine prostate. H&E staining of transverse sections of prostate ventral, lateral, dorsal, and anterior lobes at 24 months of age. Hyperplastic epithelial lesions indicated by blue arrows, stromal inflammation indicated by black arrows. Original magnification, 5×, inset 40×. Scale bars indicate 400 µm in 10×, 50 µm in 40×.

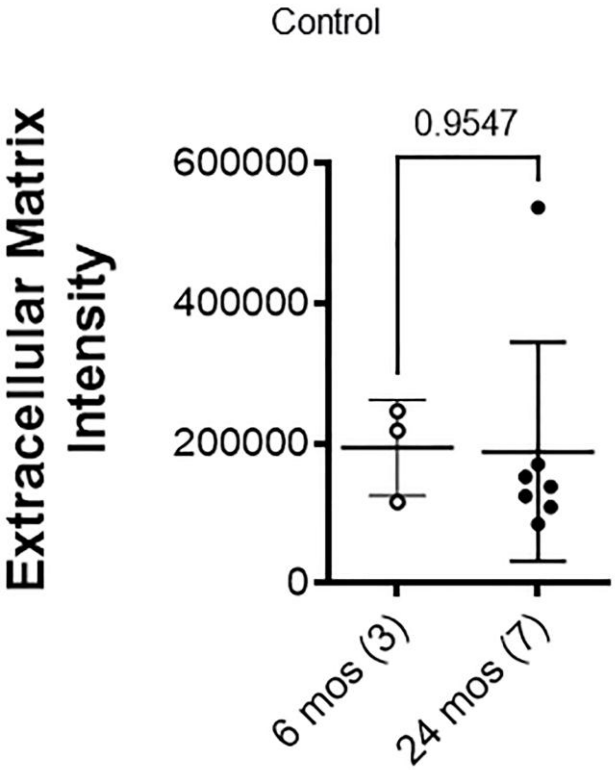

Supplementary Figure 4. Impact of aging on extracellular matrix in control mice. Quantification of Masson's trichrome staining of extracellular matrix in the stroma surrounding prostate glands from ventral prostate of Control mice at 6 months and 24 months of age. Seven fields from each section were analyzed and an average score was determined for each mouse. Data represent mean ± S.D, number of mice in each group in parentheses.
